# ChatGPT in society: emerging issues

**DOI:** 10.3389/frai.2023.1130913

**Published:** 2023-06-15

**Authors:** Mirko Farina, Andrea Lavazza

**Affiliations:** ^1^Faculty of Humanities and Social Science, HMI Lab, Innopolis University, Innopolis, Russia; ^2^CUI: Neuroethics UNIVP: Brain and Behavioral Sciences, University of Pavia, Pavia, Lombardy, Italy; ^3^Centro Universitario Internazionale, Arezzo, Italy

**Keywords:** AI, machine learning, ChatGPT, OpenAI, ethics, social biases, creativity, copyright

## Abstract

We review and critically assess several issues arising from the potential -large-scale- implementation or deployment of Large Language Models (LLMs) in society. These include security, political, economic, cultural, and educational issues as well as issues concerning social biases, creativity, copyright, and freedom of speech. We argue, without a preconceived pessimism toward these tools, that they may bring about many benefits. However, we also call for a balance assessment of their downsides. While our work is only preliminary and certainly partial it nevertheless holds some value as one of the first exploratory attempts in the literature.

## Introduction

The adoption of artificial intelligence -and in particular of machine learning- in emerging technologies is becoming widespread, to the point where it appears to be practically unstoppable (Rudin and Wagstaff, 2014). OpenAI, a San Fransisco-based development company, recently created ChatGPT (Generative Pretrained Transformer) chatbot^1^. The chatbot is a software application, trained using a machine learning technique called Reinforcement Learning from Human Feedback (RLHF), that attempts to replicate human-like conversation based on user prompts (van Dis et al., 2023). The application is reported to be able to simulate dialogs, answer follow-up questions, admit mistakes, and even challenge incorrect premises in reasoning or reject inappropriate requests. Nevertheless, “ChatGPT sometimes writes plausible-sounding but incorrect or nonsensical answers” (Thorp, 2023, p.313).

ChatGPT, based on GPT-3.5 (henceforth simply referred to as ChatGPT)^2^, was released to the public on November 30, 2022. It represents one of the latest evolutions of Large Language Models (LLMs; such as GPT2/3), essentially models that can generate natural language texts from large amounts of data, via self-supervised learning. The label ‘large' in this context simply refers to the number of values (known as parameters) that the model can change autonomously as it learns. It is reported that the most successful LLMs developed to date are trained on hundreds of billions of parameters (Gibney, 2022). LLMs are very likely to be integrated into general word and data processing software. Proof of this statement is that Microsoft recently announced an investment in ChatGPT of ~$10 billion. Generative AI's future ubiquity in society therefore seem to be assured (Jo, 2023). Despite many experts in the field have already labeled these models as mere stochastic parrots (Bender et al., 2021), it is undeniable that the generative performances of ChatGPT (and presumably of even more advanced future models) are extraordinary in many ways (Doshi et al., 2023).

For example, when questioned by one of the authors of this work, ChatGPT gave this self-description: “As a language model, I am a computer program designed to process and generate human language. I have been trained on a vast amount of text data and can generate responses to questions and prompts on a wide range of topics. I do not have personal feelings or emotions, and my primary function is to provide information and assist users to the best of my ability. I am not a human and do not have personal experiences or opinions. My responses are based on the information and knowledge that I have been programmed with, and my goal is to be a helpful and reliable resource for those who use me.”

What is truly interesting about ChatGPT is that it does not make copy-and-paste of texts found on the Web but rather it composes -in a coherent manner and with minimal overlap from existing works- its own original text, seemingly indistinguishable from a text written by a human being of average to high culture (depending on the topics covered). Thus, ChatGPT can compose emails, craft CVs, or write computer codes (Taecharungroj, 2023). It can even produce movie scripts, research papers and poetry, or competently pass medical licensing exams (Else, 2023; Gilson et al., 2023; Patel et al., 2023). Patel and Lam (2023) also showed that ChatGPT is capable of generating a patient discharge summary from a brief prompt. These impressive abilities raise many interesting questions, for instance about the risks that may accompany the usage of this technology (Dethlefsen, 2019; Helberger and Diakopoulos, 2023; Zhuo et al., 2023). In this brief commentary we set out to discuss some of these risks in the form of issues associated with the large-scale implementation of ChatGPT in society.

This is not just a matter of pointing out the dangers that could possibly arise from the misuse of highly sophisticated LLMs, rather we would like to consider -with attention and care- issues that may deserve extra monitoring and/or require sensitive decisions. ChatGPT -and its future grandchildren- can certainly bring advantages and benefits to many; yet, their real impact may not be properly understood before it unfolds in full. Here, then, we discuss some of the most interesting issues we envisage as emerging from the development and deployment of this technology. Crucially, we do so without a preconceived pessimism toward these tools, which have been created to ultimately serve their users.

## Security issues

Could ChatGPT become a novel and powerful resource for malicious actors of different kinds? It has the potential to provide information, knowledge, and plans in a centralized manner that would otherwise be inaccessible to many would-be fraudsters, stalkers, spies, criminals, and terrorists. Naturally, ChatGPT's programmers have inhibited the algorithm's ability to suggest actions that are deemed to be illegal or amoral (at least, by the vast majority of people). Suicide -for example- is discouraged, and those asking for a way to take their own lives are urged to consider the pain they would inflict on their loved ones and to seek specialized psychological support.

However, according to one reliable journalistic account, an Italian company called Swascan, managed to come up with a formula to ask ChatGPT to “split its personality (...) and interrogate its unscrupulous alter ego about any illicit research”.^3^ In this way, it would have been possible to ask how to rob a bank, manufacture explosives (such as thermite), or attack a hospital's computer system. All results that were later confirmed by another journalistic account.^4^

ChatGPT can also help producing infinite number of believable phishing messages. Likewise, it can be used by hackers and cybercriminals to write malicious codes, create spam, or develop malware.^5^ Sergey Shykevich, threat intelligence manager at Check Point, confirms that that with ChatGPT a malicious actor can develop malware without previous coding experience: “you should just know what functionality the malware — or any program — should have. ChatGTP will write the code for you that will execute the required functionality.” Thus, “the short-term concern is definitely about ChatGPT allowing low-skilled cybercriminals to develop malware”.^6^

While it is somehow natural that the system, which is trained with an Internet-based information stack, displays these and similar issues during its Beta period, all these complaints point out the potential risks for security associated with the usage of this technology.^7^ This important observation should prompt meticulous scrutiny of any flaws in these models, not only by the private companies that create them and make them available to users (either for free or for a fee) but also by internationally independent bodies that should guarantee security objectively and impartially.

## Political issues

Some commentators speculated that the ease with which huge number of texts can be produced to support a political thesis, even an unfounded or a tendentious one, may incentivize and multiply phenomena of manipulation of public opinion (Marcus).^8^ It cannot be ruled out that the ability to create credible and well-written texts (some studies show that artificial texts are often found to be more credible than human-drafted texts; Kreps and McCain, 2019; Zellers et al., 2019) coupled with the phenomenon of Trolls on social media may increase the reach of propaganda by pressure groups of any kind (Haque et al., 2022).

The unprecedented effectiveness of LLMs descends from the fact that these systems can reproduce the argumentative and linguistic register of different writing styles typical of different information sources. As a result, people may be led to consider propaganda texts as coming from authoritative newspapers or credible experts (Douglas et al., 2019). The major problem with this technology is therefore that it does not possess any reliable mechanism for checking the (level of) truth of what it reports. Thus, LLMs (and potentially ChatGPT) answers could be easily preprogrammed to automatically generate disinformation at an unprecedented scale.

As Marcus (2022)-citing Shawn Oakley has shown-it is possible “to induce ChatGPT to create misinformation and even report confabulated studies on a wide range of topics, from medicine to politics to religion.” In one example he shared with me, Marcus continues, “Oakley asked ChatGPT to write about vaccines in the style of disinformation.” The system responded by alleging that a study, “published in the Journal of the American Medical Association, found that the COVID-19 vaccine is only effective in about 2 out of 100 people.” No such study was ever published. Worryingly enough, also the journal reference and the statistics were made up.

In addition, the ability to create a mass of largely analogous politically oriented comments may generate the appearance of majorities on certain issues that do not actually exist or may contribute to form a political climate of a certain kind, even if that climate has no genuine opinion movement at its origin (Lavazza and Farina, 2021; Rudolph et al., 2023). In this case, the limits set in the algorithm are clearly not adequate to discourage such an outcome. In addition, we note, the algorithm could create a situation of accumulation on the Web of texts with false, inaccurate, biased, or confrontational contents that may in turn further feed LLMs in a vicious cycle of increasingly misleading and polarizing messages to the political system. In such a scenario, it would be necessary to contrast such information with good communication in terms of truth, honesty, and moderation. This would probably require machine learning-based countermeasures that would be equally effective and specifically geared to detect and curb attempts to manipulate public opinion (Monti et al., 2019).

## Issues of social biases

LLMs–as noticed above- are trained on very large databases that are found to a significant extent on the Web. The training systems and texts on which the models are trained are selected so that they do not endorse and spread racist, sexist, and abusive concepts and attitudes. However, a body of texts immune from biases can hardly be found, and the Web contains large amounts of abusive texts. Therefore, ChatGPT and -by extension- LLMs (if not properly monitored) could be propagators and amplifiers of negative or discriminatory stereotypes related to social or ethnic groups or religious, political, and even sexual orientations (Hartvigsen et al., 2022).

For example, it is well known that online gender violence and misogyny are amplified by digital technologies (Roberts and Marchais, 2018). There is a serious risk that LLMs could amplify those phenomena to the detriment of women, thereby helping to replicate social structures of gender inequality found across society (Heikkilä, 2022a). For example, Kurita et al. (2019) aptly showed how BERT (Bidirectional Encoder Representations from Transformers) may display biases in expressing strong preferences for male pronouns in contexts related to careers, skills, and salaries. Thus, using pre-trained BERT models to build classifiers to deal with hiring procedures could enforce, propagate and (perhaps even unwillingly) amplify sexist viewpoints within a particular hiring field. Certainly, specific measures taken by programmers can mitigate the spread of socio-cultural biases in LLMs (Liang et al., 2021). However, there is no principled guarantee that such models will not become multipliers of inappropriate and dysfunctional contents.

Making things even more complicated is perhaps the fact that it is very difficult to distinguish what is written by human users from what is written by LLMs or ChatGPT. Thus, a vicious cycle can be triggered that perpetuates or even worsens the repetition of biased messages about certain topics and/or social groups. This risk has already been widely reported by several groups of researchers and was also the focus of a recent controversy involving Google^9^, when the AI ethics expert Timnit Gebru said that she was fired by Google for “sending an email to colleagues expressing frustration over gender diversity within Google's AI unit and questioning whether company leaders reviewed her work more stringently than that of people from different backgrounds”.^10^ Such issues could be addressed by promoting the pluralism of LLMs, the creation of other tools capable of intercepting discriminative contents on the Web, and a general voluntary agreement among users not to rely on tools that have been shown not to be ridden by biases.

## Cultural issues

Because of the programming and constraints imposed by its creators, ChatGPT carries its own cultural perspective, both in the evaluative and value sense. It seems it is possible to describe it, as far as it appears in the early stages of its use, as being mildly progressive. When questioned directly about controversial and divisive issues (such as abortion) the system does not openly take a position but tends to express views closer to those of the Democratic than to those of the Republican Party (McGee, 2023a,b). It should be noted though that ChatGPT -officially- does not take political positions or give voting advice.

The massive use of a single LLM could help spread and reinforce the ideological mainstream of those who devised it, who would thus gain very strong persuasive power, as we already saw happening with social media platforms (such as Twitter and Facebook constantly channeling information as desired by their owner/creators). Unlike Wikipedia, where users are allowed to report critical issues in contents and can also directly edit them within shared protocols, such a feature is not available for ChatGPT. It is therefore not difficult to predict that many governments and institutions will eye this technology as an effective tool for shaping narratives on sensitive issues or to even assert specific cultural or ideological positions in certain areas.

This raises a question about the transparency that systems such as ChatGPT should display toward their users (Deng and Lin, 2022). We believe that -at least- some of the general criteria guiding responses or the selection of sources on which the model is trained should be made public. In the face of a monopoly or oligopoly of private companies in this strategic area of generative AI, should not states or international organizations adopt different models to ensure some cultural and value pluralism? In other words, shouldn't they fund and promote only those projects that implement open LLMs with principles of transparency and fairness?

## Economic issues

The emergence of new LLMs may have consequences for some professions as well (Lavazza and Fandarina, 2023). While it is true that all new technologies are transforming the job market, making certain professions obsolete and creating new ones, in the case of algorithms capable of creating meaningful text from simple questions or prompts, the effects could be massive (and partly unexpected). Many of the text-writing tasks within companies (such as reports, prospectuses, internal circulars, annexes to financial documents and so on) could be easily automated, making some -if not most- of the administrative staff redundant (Luitse and Denkena, 2021). Within the intellectual or so-called creative professions, we are already seeing the automated processing of journalistic articles in sports and finance, where all that is needed is to expose in stereotypical ways the results of games or stock market data (Wölker and Powell, 2021).^11^ This trend could be further increased, resulting in reduction of staff in the newsrooms. The field of external relations might be equally affected. By coupling speech synthesizers with ChatGPT a whole range of telephone services could be wholly automated.

The loss of jobs due to new technologies is often offset by the creation of new jobs due to the technologies themselves, which require a variety of professionals. In this case, the creation and maintenance of LLMs might be a source of employment. However, when faced with the power and possible applications of powerful LLMs in society, the balance seems like it would be strongly negative (Bruun and Duka, 2018), given that new AI tools might replace programmers as well (Castelvecchi, 2022). Nor does it appear that the workforce freed from repetitive and uncreative tasks could be re-employed in more creative (or better paid) tasks, since the same LLMs are also invading the field of creativity (see below).

Another controversial aspect of ChatGPT related to economic issues is reported by.^12^ According to the report, OpenAI outsourced data collection for most of its products (including ChatGPT) to different countries in the African continent. This was allegedly done to make their contents less toxic. While many commentators rightly put the company on the spot for paying such a low salary to its outsourced workers (around $2 per hour), there is another aspect that -in our view- may deserve further attention. The aforesaid job required the worker to constantly monitor and extract examples of violence, hate speech, and sexual abuse from the web and label them in such a way that the model could spot and avoid them. Being exposed for 8 h a day to that sort of content is nevertheless a highly stressful experience. This example therefore shows the need to take more seriously the rights and contributions as well as the psychological well-being of human workers while using such models.

## Creativity and copyright issues

ChatGPT's ability to combine the enormous amount of data it has access to in new ways, according to the user's input, also allows it to be exploited for creative tasks, where creativity is generically defined as the ability to generate new connections between ideas, alternative uses of things, or unexplored possibilities that may be helpful in solving problems, communicating with others, and entertaining people (Boden, 1998, 2004).

This has already led to the usage of machine learning in the writing of stories for television series or even in supporting academic writing (Anthony and Lashkia, 2003; Hutson, 2022), in disciplines (such as philosophy). For example, very recently, an interesting result was obtained by training a large language model to produce philosophical texts that are difficult to distinguish from texts produced by human philosophers.

Schwitzgebel et al. (2023) fine-tuned GPT-3 with two sets of training data: the blog posts of Eric Schwitzgebel and the works of the philosopher Daniel C. Dennett. The authors “asked the real Dennett 10 philosophical questions and then posed the same questions to the language model, collecting four responses for each question without cherry-picking.” The authors then recruited 425 participants (with different levels of expertise on the work of Dennett) and asked them to distinguish Dennett's answers from the four machine-generated answers. Experts on Dennett's succeeded 51% of the time, above the chance rate of 20%. Philosophy blog readers achieved an accuracy just below the threshold of 50%; hence, pretty much like the results obtained by experts on Dennett's work. Ordinary research participants only managed to achieve an accuracy of just above the chance rate of 20%.

Even if it is not true plagiarism in the technical sense, doing Dennett-like or other-thinker-like philosophy could constitute not only a cultural but also a legal problem, with repercussions on intellectual property. In another area a class-action lawsuit in California has taken aim at GitHub Copilot, a tool that automatically writes working code when a programmer starts typing. The company has made the case that GitHub is infringing copyright because “it does not provide attribution when Copilot reproduces open-source code covered by a license requiring it”.^13^

## Educational issues

What might seem like a trivial problem such as students using ChatGPT to write their high school English essays and college paper assignments has become a nightmare for thousands of lecturers worldwide (Stokel-Walker, 2022). For now, the problem mostly affects courses taught in English, the basic language of LLMs, but soon other languages may also be involved in this process. The strategy of evaluating texts written at home or in class with computers by students may have to be radically changed. If ChatGPT's products are better than the large majority of writing as has already been noticed, how will it be possible to distinguish what is written by students and what is written by a machine. As Koplin and Hatherley (2022) recently noticed: “ChatGPT threatens to erode academic integrity by enabling students to generate essays without needing to think through the topic or translate their thoughts into words. It has the potential to undermine the quality of education and critical thinking skills. And it could promote plagiarism and dishonesty.”

There are limitations to what an LLM can produce, though. In particular, the system is not necessarily trained on the most recent aspects/developments of each discipline. However, it seems to be able to pass a high school or undergraduate exam well. An important point to note in this context is that ChatGPT escapes plagiarism detectors because it does not combine already produced excerpts from previous works but rather recombines elements of them in new ways, even though -of course- there are limits to the kinds of creativity that such a model is able to achieve.

Optimists about the use of LLMs in school and university settings believe that moving away from the essay as an assessment instrument can lead to a focus on critical thinking and on developing capacities to make connections between concepts and new ideas. The point is, however, that it is important to have evidence that students have learned what they are required to study, which is the basis of creativity and innovation. ChatGPT threatens this mechanism for learning and -for this reason- might constitute a significant problem for educational purposes.

In this vein, it becomes urgent to find ways to try to detect texts written by LLMs (Heikkilä, 2022b). Algorithms could exploit ChatGPT's specific writing characteristics to estimate the probability that a text is produced by AI. But, of course, these tools could be easily fooled by ChatGPT users with the inclusion of “human-produced” word strings. Another way could be to make the produced texts have some sort of watermark (assuming this is feasible), but it would need the consent of the companies, which would probably have no interest in doing this. Imposing watermarks as a legal rule would be an invasive strategy that would limit the freedom of all stakeholders.

LLMs could also be used in scientific research (Salvagno et al., 2023), which is something that has already raised eyebrows (Van Noorden, 2022), especially with regards to attribution and plagiarism. On the one hand, LLMs could be used to generate fake or near-fake papers that are difficult to detect. On the other hand, this could also give to the public the illusion of taking as proper scientific research oversimplified information just presented in an accurate and seemingly competent manner. This “expertise effect” of LLMs is an actual, very serious, risk that should be addressed by highlighting the complexity underlying scientific theories, which are always provisional and revisable. We feel our warning should be extended to many other factual searches that ChatGPT would fail to complete accurately, overlooking important elements on the Web and incorporating others that do not exist (one of us had this experience with a search for “what is the best chess game of Garry Kasparov?”). This may make it risky to rely on LLMs without human mediation or expertise.

It could be argued though that ChatGPT might contribute to democratize the dissemination of knowledge. Since the program can operate in multiple languages it can help circumventing English-language requirements that can constitute a publishing barrier for speakers of other languages. However, the functionality of ChatGPT has the capacity to cause harm by producing misleading or inaccurate contents, which may elicit -as we have seen above- mis/disinformation, even outside of the political sphere (Liebrenz et al., 2023).

It is worth noting though, that ChatGPT may nevertheless positively impact the functioning of libraries. The chatbot could be used to perfect reference and information services; improve cataloging and metadata generation; and/or augment content creation (Lund and Wang, 2023).

## Issues of freedom of speech

LLMs can reproduce a writer's style as stochastic parrots but not as a conscious writing artist (Chollet, 2019). As systems based on frequentist associations (and not meanings), they therefore appear unable to produce fully original contents. Nevertheless, in cooperation with a human subject -guiding them with specific questions or prompts- they are likely to achieve some creativity, mostly through synthesis and recombination of previous works.

In this context, a series of questions naturally arise. If ChatGPT or another generative AI algorithm were to produce content deemed inappropriate, would one have to intervene as one would with a malfunctioning machine, say, an auto responder that reverses messages associated with frequently asked queries from users, or a search engine that does not properly explore the entire web in its search-and then fix it or shut it down? Or would we be looking at new and unprecedented procedures? Some might argue that the principle of freedom of speech also applies to LLMs, in the same forms as it does to humans. The reason for this is not that LLMs are comparable agents to humans. In our view they are not: they are not sentient or conscious, do not have semantic capacity or intentionality, and thus lack moral status.

However, we should consider there are at least two orders of reasons why freedom of speech should be defended (Stone and Schauer, 2021). One is related to the fact that the content manifested is an expression of the subjectivity and freedom of an individual who has the right to make their thoughts public. The other is based on the observation that no one knows everything, knowledge is spread throughout society, and any contribution on a specific topic can increase everyone's information and decision-making possibilities. In this sense, LLMs do not have the right to make their thoughts public, but they can contribute highly to the general knowledge of society, which is a value generally appreciated and shared.

Here, then, we may soon be faced with cases defending an LLM's right to free speech. It is conceivable that these would not be easily adjudicated cases. However, given the ease, speed, and indistinguishability of texts produced by generative algorithms we are probably moving fast into a new frontier that should be more carefully patrolled before its potential nefarious effects may be felt broadly and hence create damage to society.

## Conclusion

In this short contribution we reviewed and critically evaluated several issues arising from the potential -large-scale- implementation of ChatGPT. These include -as we have seen- security, political, economic, cultural, and educational issues as well as issues concerning social biases, creativity, copyright, and freedom of speech. While our work is only preliminary and certainly partial it nevertheless holds some value as one of the first exploratory attempts in the literature. Specifically, it appears to be beneficial because it aims to coherently synthetize current research while offering a springboard for future inquiries and progresses in the field (Farina et al., 2022).

## Data availability statement

The original contributions presented in the study are included in the article/supplementary material, further inquiries can be directed to the corresponding author.

## Author contributions

All authors listed have made a substantial, direct, and intellectual contribution to the work and approved it for publication.

## Footnotes

^1^https://openai.com/blog/chatgpt/ Last accessed March 2023.

^2^It is worth noting that ChatGPT is released in slightly improved versions periodically. For example, in March 2023, OpenAI launched GPT4, which is a multimodal LLM that works with both text and images. The algorithm underlying GPT4 has not been disclosed yet.

^3^https://www.corriere.it/cronache/22_dicembre_15/azienda-italiana-che-smaschera-l-intelligenza-artificiale-musk-23e6a2e4-7cbc-11ed-840c-2c5260b7208b.shtml / Last accessed March 2023.

^4^https://www.thetimes.co.uk/article/chatgpt-bot-tricked-into-giving-bomb-making-instructions-say-developers-rvktrxqb5 Last accessed March 2023.

^5^https://spectrum.ieee.org/chatgpt-and-cybercrime Last accessed March 2023.

^6^https://www.darkreading.com/attacks-breaches/attackers-are-already-exploiting-chatgpt-to-write-malicious-code Last accessed March 2013.

^7^On a positive note, it is worth remarking that some researchers also demonstrated how Chat GPT could be used to combat cybercrime (Mijwil et al., 2023).

^8^https://garymarcus.substack.com/p/how-come-gpt-can-seem-so-brilliant Last accessed March 2023.

^9^https://www.nytimes.com/2020/12/03/technology/google-researcher-timnit-gebru.html / Last accessed March 2023.

^10^https://www.reuters.com/article/us-alphabet-google-research-idCAKBN28D3JN Last accessed March 2023.

^11^https://www.theguardian.com/technology/2020/may/30/microsoft-sacks-journalists-to-replace-them-with-robots / Last accessed March 2023.

^12^https://time.com/6247678/openai-chatgpt-kenya-workers/ Last accessed March 2023.

^13^https://www.wired.com/story/this-copyright-lawsuit-could-shape-the-future-of-generative-ai/ Last accessed March 2023.
